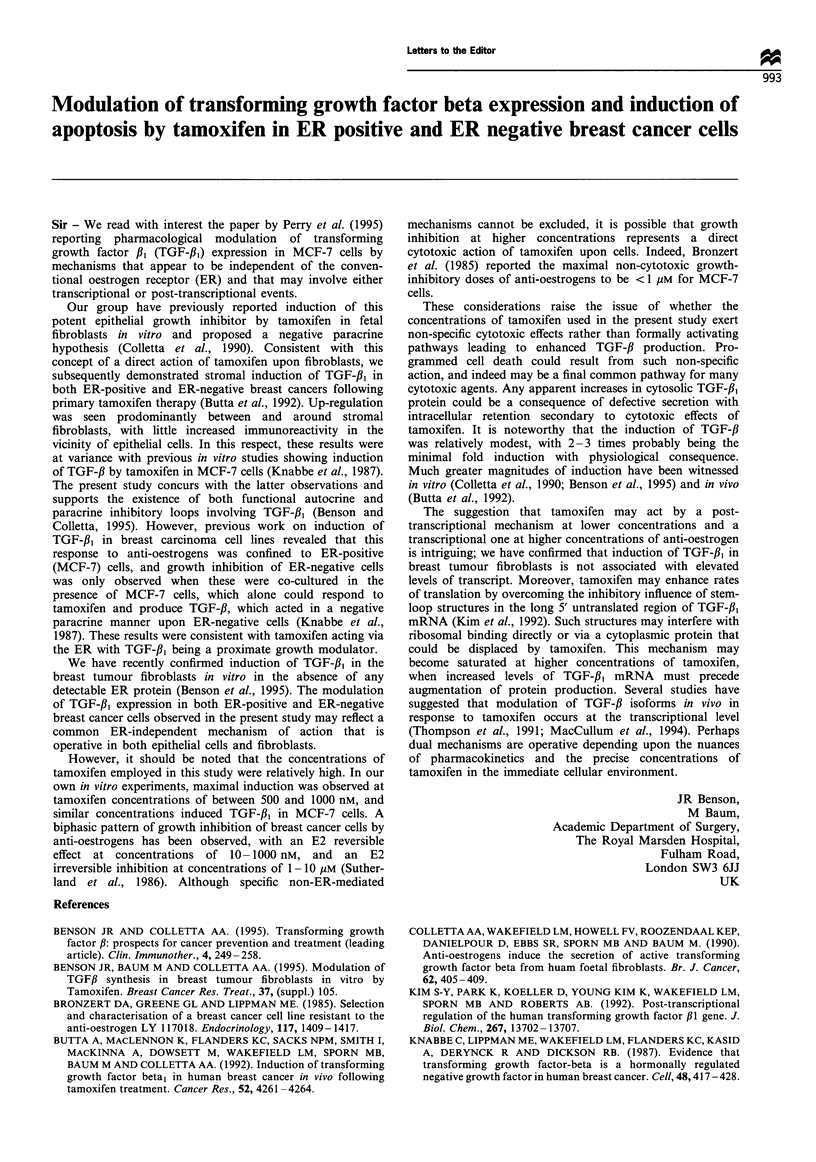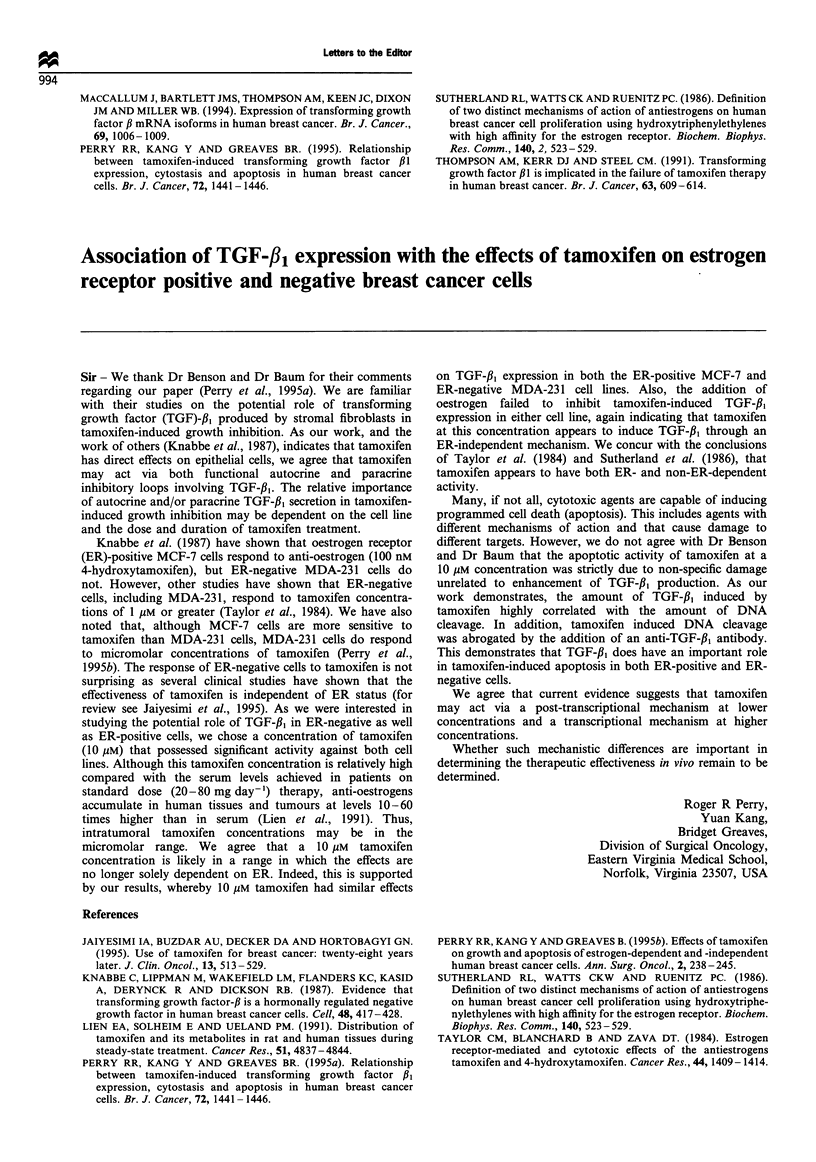# Modulation of transforming growth factor beta expression and induction of apoptosis by tamoxifen in ER positive and ER negative breast cancer cells.

**DOI:** 10.1038/bjc.1996.475

**Published:** 1996-09

**Authors:** J. R. Benson, M. Baum


### Modulation of transforming growth factor beta expression and induction of apoptosis by tamoxifen in ER positive and ER negative breast cancer cells

**Sir** – We read with interest the paper by Perry *et al.* (1995) reporting pharmacological modulation of transforming growth factor $\beta_1$ (TGF-$\beta_1$) expression in MCF-7 cells by mechanisms that appear to be independent of the conventional oestrogen receptor (ER) and that may involve either transcriptional or post-transcriptional events.

Our group have previously reported induction of this potent epithelial growth inhibitor by tamoxifen in fetal fibroblasts *in vitro* and proposed a negative paracrine hypothesis (Colletta *et al.*, 1990). Consistent with this concept of a direct action of tamoxifen upon fibroblasts, we subsequently demonstrated stromal induction of TGF-$\beta_1$ in both ER-positive and ER-negative breast cancers following primary tamoxifen therapy (Butta *et al.*, 1992). Up-regulation was seen prodominantly between and around stromal fibroblasts, with little increased immunoreactivity in the vicinity of epithelial cells. In this respect, these results were at variance with previous *in vitro* studies showing induction of TGF-$\beta$ by tamoxifen in MCF-7 cells (Knabbe *et al.*, 1987). The present study concurs with the latter observations and supports the existence of both functional autocrine and paracrine inhibitory loops involving TGF-$\beta_1$ (Benson and Colletta, 1995). However, previous work on induction of TGF-$\beta_1$ in breast carcinoma cell lines revealed that this response to anti-oestrogens was confined to ER-positive (MCF-7) cells, and growth inhibition of ER-negative cells was only observed when these were co-cultured in the presence of MCF-7 cells, which alone could respond to tamoxifen and produce TGF-$\beta$, which acted in a negative paracrine manner upon ER-negative cells (Knabbe *et al.*, 1987). These results were consistent with tamoxifen acting via the ER with TGF-$\beta_1$ being a proximate growth modulator.

We have recently confirmed induction of TGF-$\beta_1$ in the breast tumour fibroblasts *in vitro* in the absence of any detectable ER protein (Benson *et al.*, 1995). The modulation of TGF-$\beta_1$ expression in both ER-positive and ER-negative breast cancer cells observed in the present study may reflect a common ER-independent mechanism of action that is operative in both epithelial cells and fibroblasts.

However, it should be noted that the concentrations of tamoxifen employed in this study were relatively high. In our own *in vitro* experiments, maximal induction was observed at tamoxifen concentrations of between 500 and 1000 nM, and similar concentrations induced TGF-$\beta_1$ in MCF-7 cells. A biphasic pattern of growth inhibition of breast cancer cells by anti-oestrogens has been observed, with an E2 reversible effect at concentrations of 10–1000 nM, and an E2 irreversible inhibition at concentrations of 1–10 $\mu$M (Sutherland *et al.*, 1986). Although specific non-ER-mediated mechanisms cannot be excluded, it is possible that growth inhibition at higher concentrations represents a direct cytotoxic action of tamoxifen upon cells. Indeed, Bronzert *et al.* (1985) reported the maximal non-cytotoxic growth-inhibitory doses of anti-oestrogens to be $<1$ $\mu$M for MCF-7 cells.

These considerations raise the issue of whether the concentrations of tamoxifen used in the present study exert non-specific cytotoxic effects rather than formally activating pathways leading to enhanced TGF-$\beta$ production. Programmed cell death could result from such non-specific action, and indeed may be a final common pathway for many cytotoxic agents. Any apparent increases in cytosolic TGF-$\beta_1$ protein could be a consequence of defective secretion with intracellular retention secondary to cytotoxic effects of tamoxifen. It is noteworthy that the induction of TGF-$\beta$ was relatively modest, with 2–3 times probably being the minimal fold induction with physiological consequence. Much greater magnitudes of induction have been witnessed *in vitro* (Colletta *et al.*, 1990; Benson *et al.*, 1995) and *in vivo* (Butta *et al.*, 1992).

The suggestion that tamoxifen may act by a post-transcriptional mechanism at lower concentrations and a transcriptional one at higher concentrations of anti-oestrogen is intriguing; we have confirmed that induction of TGF-$\beta_1$ in breast tumour fibroblasts is not associated with elevated levels of transcript. Moreover, tamoxifen may enhance rates of translation by overcoming the inhibitory influence of stem-loop structures in the long 5′ untranslated region of TGF-$\beta_1$ mRNA (Kim *et al.*, 1992). Such structures may interfere with ribosomal binding directly or via a cytoplasmic protein that could be displaced by tamoxifen. This mechanism may become saturated at higher concentrations of tamoxifen, when increased levels of TGF-$\beta_1$ mRNA must precede augmentation of protein production. Several studies have suggested that modulation of TGF-$\beta$ isoforms *in vivo* in response to tamoxifen occurs at the transcriptional level (Thompson *et al.*, 1991; MacCullum *et al.*, 1994). Perhaps dual mechanisms are operative depending upon the nuances of pharmacokinetics and the precise concentrations of tamoxifen in the immediate cellular environment.

JR Benson,
M Baum,
Academic Department of Surgery,
The Royal Marsden Hospital,
Fulham Road,
London SW3 6JJ
UK

### References

BENSON JR AND COLLETTA AA. (1995). Transforming growth factor $\beta$: prospects for cancer prevention and treatment (leading article). *Clin. Immunother.*, **4**, 249–258.

BENSON JR, BAUM M AND COLLETTA AA. (1995). Modulation of TGF$\beta$ synthesis in breast tumour fibroblasts in vitro by Tamoxifen. *Breast Cancer Res. Treat.*, **37**, (suppl.) 105.

BRONZERT DA, GREENE GL AND LIPPMAN ME. (1985). Selection and characterisation of a breast cancer cell line resistant to the anti-oestrogen LY 117018. *Endocrinology*, **117**, 1409–1417.

BUTTA A, MACLENNON K, FLANDERS KC, SACKS NPM, SMITH I, MACKINNA A, DOWSETT M, WAKEFIELD LM, SPORN MB, BAUM M AND COLLETTA AA. (1992). Induction of transforming growth factor beta$_1$ in human breast cancer *in vivo* following tamoxifen treatment. *Cancer Res.*, **52**, 4261–4264.

COLLETTA AA, WAKEFIELD LM, HOWELL FV, ROOZENDAAL KEP, DANIELPOUR D, EBBS SR, SPORN MB AND BAUM M. (1990). Anti-oestrogens induce the secretion of active transforming growth factor beta from huam foetal fibroblasts. *Br. J. Cancer*, **62**, 405–409.

KIM S-Y, PARK K, KOELLER D, YOUNG KIM K, WAKEFIELD LM, SPORN MB AND ROBERTS AB. (1992). Post-transcriptional regulation of the human transforming growth factor $\beta$1 gene. *J. Biol. Chem.*, **267**, 13702–13707.

KNABBE C, LIPPMAN ME, WAKEFIELD LM, FLANDERS KC, KASID A, DERYNCK R AND DICKSON RB. (1987). Evidence that transforming growth factor-beta is a hormonally regulated negative growth factor in human breast cancer. *Cell*, **48**, 417–428.

MACCALLUM J, BARTLETT JMS, THOMPSON AM, KEEN JC, DIXON JM AND MILLER WB. (1994). Expression of transforming growth factor $\beta$ mRNA isoforms in human breast cancer. *Br. J. Cancer.*, **69**, 1006–1009.

PERRY RR, KANG Y AND GREAVES BR. (1995). Relationship between tamoxifen-induced transforming growth factor $\beta 1$ expression, cytostasis and apoptosis in human breast cancer cells. *Br. J. Cancer*, **72**, 1441–1446.

SUTHERLAND RL, WATTS CK AND RUENITZ PC. (1986). Definition of two distinct mechanisms of action of antiestrogens on human breast cancer cell proliferation using hydroxytriphenylethylenes with high affinity for the estrogen receptor. *Biochem. Biophys. Res. Comm.*, **140**, *2*, 523–529.

THOMPSON AM, KERR DJ AND STEEL CM. (1991). Transforming growth factor $\beta 1$ is implicated in the failure of tamoxifen therapy in human breast cancer. *Br. J. Cancer*, **63**, 609–614.